# AP-1 Gene Expression Levels May Be Correlated with Changes in Gene Expression of Some Stemness Factors in Colon Carcinomas

**DOI:** 10.1155/2013/497383

**Published:** 2013-12-11

**Authors:** Panagiotis Apostolou, Maria Toloudi, Eleni Ioannou, Marina Chatziioannou, Eleni Kourtidou, Ioanna Vlachou, Ioannis Papasotiriou

**Affiliations:** Research Genetic Cancer Centre Ltd. (RGCC Ltd.), Filotas, 53070 Florina, Greece

## Abstract

The AP-1 transcription factor is a heterodimer protein that regulates gene expression in response to a variety of extrinsic stimuli through signal transduction. It is involved in processes including differentiation, proliferation, and apoptosis. Among the genes it regulates are transcription factors that contribute to the stemness phenotype. Cancer stem cells have the ability to self-renew and initiate differentiation into heterogenic cancer cells, which may cause metastasis and relapses. In the present study, we evaluated the effect of AP-1 complexes, as well as the *C-FOS* and *C-JUN* genes, in relation to NANOG, OCT3/4, and SOX2 transcription factors. All assays were undertaken with colon cancer stem cells. Knockdown experiments with siRNA were performed for each individual gene as well as their combination. Changes in gene expression were calculated with quantitative polymerase chain reaction experiments, while the effect on cell cycle distribution and apoptosis was studied by flow cytometry. The results differed depending on the percentage of repression, as well as the gene that was suppressed. In all cases, the number of apoptotic cells was increased. These findings indicate that AP-1 may have a crucial role in the maintenance of cancer stem cells.

## 1. Introduction

The AP-1 transcription factor consists of various proteins including *C-FOS* and C-JUN. Its function is to regulate gene expression in response to many stimuli, and it is involved in multiple cellular processes, such as differentiation, proliferation, and apoptosis [1, 2]. The monomers of the AP-1 complex are encoded by different genes. These transcription factors are located downstream many transduction pathways, thus making their role critical [3, 4]. Cancer stem cells (CSCs) are cells that are defined by their ability to self-renew and undergo asymmetric cell division, proliferation, and differentiation. With respect to their origin, these cells may be caused by disturbance of the self-renewal and differentiation programs occurring in multipotential stem cells, tissue-specific stem cells, progenitor cells, mature cells, and cancer cells [5]. The hallmarks of the CSC phenotype are defined by many genes; however, NANOG, *POU5F1* (OCT3/4), and SOX2 have crucial roles [6, 7].

Recent experimental data indicated that C-JUN is important for the maintenance of the self-renewal and tumorigenicity of glioma stem-like cells [8]. According to another study in colon cancer, C-JUN and TCF4 promoted a subpopulation of colorectal cancer tumor cells to adopt a stem-like phenotype via the *NANOG* promoter [9]. Moreover, *C-FOS* maintains hematopoietic stem cells in quiescence [10]. The present study aimed to identify the relationship between the AP-1 complex and stemness transcription factors. We attempted to address whether the AP-1 transcription factor is necessary to activate or suppress NANOG, OCT3/4, and SOX2 transcription factors as well as if it has an effect on apoptosis and the cell cycle.

## 2. Materials and Methods

### 2.1. Cell Culture

Human colon cancer stem cells (36112-39P; Celprogen) were cultured in appropriate growth medium (M36112-39PS; Celprogen), supplemented with 10% FBS in 25 cm^2^ flasks (E36102-29P-T25; Celprogen) at 37°C in a 5% CO_2_ environment.

### 2.2. Knockdown

During the exponential phase of proliferation, cells were seeded in 24-well plates (E36112-39; Celprogen) and transfected with small interfering RNA (siRNA) specific for *C-JUN* and *C-FOS* genes using Lipofectamine 2000 (11668-027; Invitrogen), according to the manufacturer's instructions. The siRNA were designed in accordance with the rules of Reynolds et al. [11], and the sequences were as follows: *C-FOS*: 5′ UAUCUGAGAAUCCAUCUUAUU 3′ and *C-JUN*: 5′ ACAUCAUGGGCUAUUUUUA 3′. All sequences were run on BLAST to exclude sequences that would suppress undesired genes and to ensure specificity. After 48 h incubation, the cells were harvested by trypsinization (25200-072; Invitrogen). Samples incubated with Lipofectamine alone (without siRNA) were also tested to study the effect of compound alone on gene expression.

### 2.3. Evaluation of Cells

Cells were tested in both cellular and molecular assays. The cellular assays were based on the ability of CSCs to form microspheres in semisuspension, using an inverted light microscope. The cultures have previously been evaluated by molecular analyses, including gene expression analysis for specific transcription factors [12]. The authentication of the control cell line was tested each time using molecular biology assays, such as short tandem repeat profiling, the results of which were compared with the manufacturer's profile. Cultivation was continued for more than 30 passages to exclude the possibility of incorporating embryonic stem cells (ESCs) in the experiments, since cancer stem cells are immortal unlike ESCs.

### 2.4. Molecular Analysis

RNA was extracted from cell cultures using an RNeasy Mini Kit (74105; Qiagen). The RNA samples were evaluated both spectrophotometrically and on agarose gel by checking the 18S-28S rRNA bands. Then, 1 *μ*g of each sample was used as template for cDNA synthesis using an iScript cDNA synthesis kit (1708891; Bio-Rad). Finally, the upper strand was used as template for real-time polymerase chain reaction (PCR), which was performed using the iTaq Universal SYBR Green Supermix (1725124; Bio-Rad). Specific primers for each marker and for an endogenous control gene (18S rRNA) were designed using Gene Expression 1.1 software. The sequence of primers was run on BLAST to exclude those that would amplify undesired genes. The sequence of the primers was as follows: *18SrRNA*: forward—5′ TGCCCTATCAACTTTCGATGGTAGTC 3′, reverse—5′ TTGGATGTGGTAGCCGTTTCTCA 3′; *NANOG*: forward—5′ TGAGATGCCTCACACGGAGACTG 3′, reverse—5′ GGGTTGTTTGCCTTTGGGACTG 3′; *POU5F1*: forward—5′ GGTGCCTGCCCTTCTAGGAATG 3′, reverse—5′ TGCCCCCACCCTTTGTGTTC 3′;* SOX2*: forward—5′ CAACGGCAGCTACAGCATGATG 3′, reverse—5′ GCGAGCTGGTCATGGAGTTGTACT 3′; *C-FOS*: forward—5′ CCTTCACCCTGCCTCTCCTCAAT 3′, reverse—5′ GCCTGGATGATGCTGGGAACA 3′; *C-JUN*: forward—5′ CCAACTCATGCTAACGCAGCAGTT 3′, reverse—5′ ACCCTTGGCTTTAGTTCTCGGACAC 3′. The PCR program was as follows: initial denaturation at 95°C are 50 cycles of denaturation at 95°C for 10 sec followed by annealing at 59°C for 30 sec. A final extension step was performed at 72°C for 10 min followed by melting curve analyses. Data were analyzed according to the Livak method [13].

### 2.5. Flow Cytometry

Cells were stained with PE Annexin V and 7-Amino-Actinomycin (7AAD) (559763; BD Biosciences) for 15 min and then resuspended in 0.5 mL sheath fluid (8546859; Beckman Coulter) followed by flow cytometric analysis of more than 50,000 events. The data were analyzed with FCS Express Software (DeNovo). In each case, positive and negative controls were used.

### 2.6. Statistical Analysis

The quantitative polymerase chain reaction (qPCR) results were tested according to the Kolmogorov-Smirnov test; all samples had normal distribution. Median values were used for the analysis. Finally, Mann-Whitney *U* tests were also performed on the qPCR data.

## 3. Results and Discussion

### 3.1. Gene Expression

**Figure 1:**
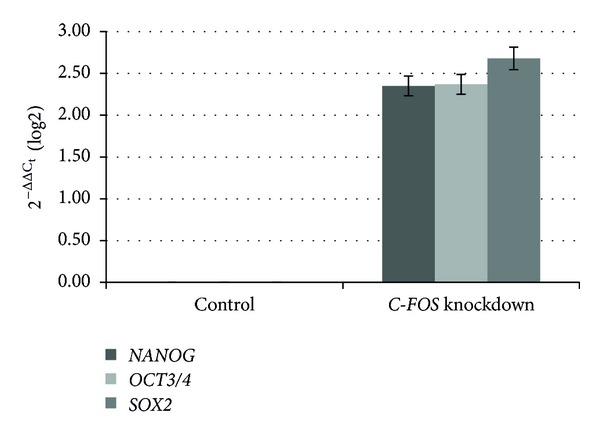
Relative gene expression analysis of stemness transcription factors, Nanog, Oct3/4, and Sox2, after *C-FOS* knockdown. Changes in gene expression caused by the suppression of *C-FOS*. The percentage of knockdown reached 55%.

**Figure 2:**
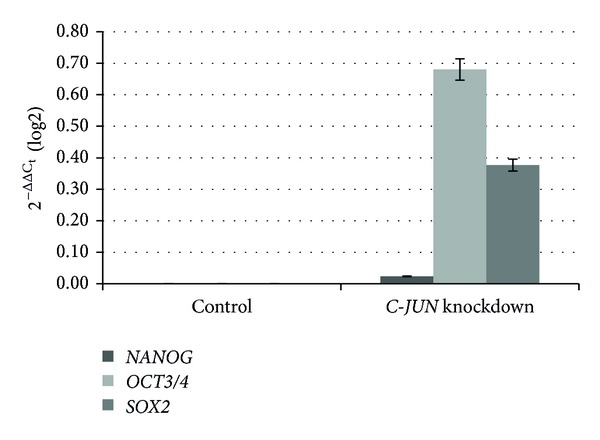
Relative gene expression analysis of stemness transcription factors, Nanog, Oct3/4, and Sox2, after *C-JUN* knockdown. Changes in gene expression caused by the suppression of *C-JUN*. The percentage of knockdown reached 30%.

**Figure 3:**
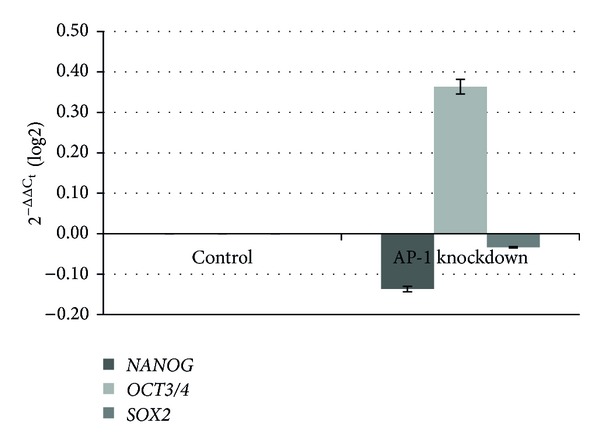
Relative gene expression analysis of stemness transcription factors, Nanog, Oct3/4, and Sox2, after AP-1 complex knockdown. Changes in gene expression caused by the suppression of AP-1. The percentage of knockdown reached 45%.

**Figure 4:**
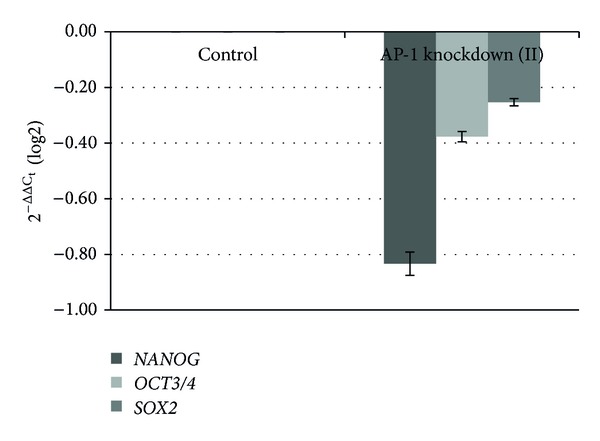
Relative gene expression analysis of stemness transcription factors, Nanog, Oct3/4, and Sox2, after AP-1 complex knockdown (II). Changes in gene expression caused by the suppression of AP-1. The percentage of knockdown reached 35%.

Knockdown was up to 55% for *C-FOS* and up to 45% when *C-FOS* and *C-JUN* were knocked down simultaneously, while lower rates were observed following knockdown of *C-JUN*. The suppression of *C-FOS *led to increases in the gene expression of stemness transcription factors. In comparison with the control cells, the increase in gene expression was 2.35-fold for *NANOG*, 2.93-fold for *POU5F1* (Oct3/4), and 2.68-fold for *SOX2*. Knockdown of *C-JUN* led to increases in *POU5F1* and *SOX2* expression of 130% and 30%, respectively, while no changes were observed in *NANOG*; this was in contrast to data resulting from simultaneous repression of *C-JUN* and *C-FOS*. The results also differed depending on the rate of reduction in gene expression. In all cases, an increase was observed in *POU5F1* expression, with decreases in the other genes. Suppression of the AP-1 complex by 45% led to a 90% increase in *POU5F1* expression and a reduction of 10% and 3% in *NANOG* and *SOX2* expression, respectively. *NANOG* expression was reduced up to 50% when knockdown reached up to 35%; *POU5F1* and *SOX2 *were less affected. Figures 1, 2, 3, and 4 graphically represent the above results.

### 3.2. Cell Cycle Distribution

The number of cells undergoing apoptosis was three times higher following suppression of both genes by 45%. Under the same conditions, it was observed that there was an increase in dead cells of 1.5 times that of the control cells. *C-FOS* knockdown only led to a doubling of dead cells; however, there was no change in the number of cells undergoing apoptosis (Table 1).

CSCs are defined by their ability to self-renew, differentiate, and proliferate. These cells are proposed to initiate tumor formation and propagate metastasis [14]. According to experimental data, there is evidence to indicate that the hallmarks of CSCs are defined by many transcription factors, but the most important are NANOG, OCT3/4, and SOX2. NANOG is expressed in ESCs and has an important role in maintaining pluripotency. Overexpression of NANOG causes self-renewal of ESCs, while its absence leads to differentiation [15–18]. To maintain stemness, the presence and, most likely, collaboration of two further transcription factors, OCT3/4 and SOX2, are required. OCT3/4 expression is also associated with the undifferentiated stage and self-renewal. It forms a heterodimer with SOX2, which binds DNA. SOX2 is a transcription factor essential for maintaining pluripotency, but its ectopic expression may be related to abnormal differentiation of colorectal cancer cells [19–21].

Although little is known about their origin, CSCs are a subpopulation of heterogeneous tumors that has the ability to enter the bloodstream and migrate to colonize secondary sites, thus resulting in metastases and relapses. The epithelial to mesenchymal transition (EMT) and the reverse process (mesenchymal to epithelial transition (MET)) contribute to this [22, 23].

The AP-1 transcription factor consists mainly of the *C-FOS* and C-JUN proteins. *C-FOS* is a protooncogene and has a leucine zipper DNA-binding domain [24]. C-JUN is also a proto-oncogene that has important roles in cellular proliferation and apoptosis [25]. The AP-1 transcription factor acts downstream many transduction pathways; therefore, many processes are implicated. Recent studies have demonstrated that C-JUN and *C-FOS* are also involved in stemness pathways. C-JUN has a pivotal role in the maintenance of self-renewal and tumorigenicity in glioma stem-like cells. In contrast, another study has implicated AP-1 and NF-*κ*B in the differentiation of mouse ESCs [8, 26–28].

Therefore, it is clear that there is a relationship between the AP-1 transcription factor and stemness. The present study aimed to clarify this relationship in colon CSCs. The contribution of the AP-1 complex has been shown in apoptosis, alongside that of the individual proteins. AP-1 seems to play a crucial role in the maintenance of stemness by controlling NANOG, OCT3/4, and SOX2. Suppression of AP-1 led to a reduction in the levels of *NANOG* and *SOX2* gene expression, which in turn led to an increase in the number of cells undergoing apoptosis. It may be that cells which cannot maintain the hallmarks of stemness eventually undergo apoptosis.

## 4. Conclusions

The present study indicated that the AP-1 transcription factor may be strongly related to the stemness phenotype in colon CSCs. The reduction of its expression leads to changes in the expression of major transcription factors that are essential for maintaining pluripotency and undifferentiation. Further studies need to be performed to further investigate this correlation.

## Figures and Tables

**Table 1:** Percentage of dead cells and cells undergoing apoptosis before and after knockdown.

Cell line	Cells undergoing apoptosis (%)	Dead cells (%)
Control	1.48	4.40
*C-FOS* knockdown	1.68	7.42
*C-JUN* knockdown	4.93	4.06
*AP-1* knockdown (45%)	6.39	5.76
*AP-1* knockdown (35%)	2.61	3.71

## Abbreviations

CSCs: Cancer stem cells
siRNA: Small interfering RNA
qPCR: Quantitative polymerase chain reaction
ESCs: Embryonic stem cells
EMT: Epithelial to mesenchymal transition
MET: Mesenchymal to epithelial transition
NF-*κ*B: Nuclear factor kappa-light-chain-enhancer of activated B cells.

## References

[1] Hess J, Angel P, Schorpp-Kistner M (2004). AP-1 subunits: quarrel and harmony among siblings. *Journal of Cell Science*.

[2] Ameyar M, Wisniewska M, Weitzman JB (2003). A role for AP-1 in apoptosis: the case for and against. *Biochimie*.

[3] Ouafik L, Berenguer-Daize C, Berthois Y (2009). Adrenomedullin promotes cell cycle transit and up-regulates cyclin D1 protein level in human glioblastoma cells through the activation of c-Jun/JNK/AP-1 signal transduction pathway. *Cellular Signalling*.

[4] Weng C-J, Chau C-F, Hsieh Y-S, Yang S-F, Yen G-C (2008). Lucidenic acid inhibits PMA-induced invasion of human hepatoma cells through inactivating MAPK/ERK signal transduction pathway and reducing binding activities of NF-*κ*B and AP-1. *Carcinogenesis*.

[5] Bu Y, Cao D (2012). The origin of cancer stem cells. *Frontiers in Bioscience*.

[6] Chiou S-H, Yu C-C, Huang C-Y (2008). Positive correlations of Oct-4 and Nanog in oral cancer stem-like cells and high-grade oral squamous cell carcinoma. *Clinical Cancer Research*.

[7] Xiang R, Liao D, Cheng T (2011). Downregulation of transcription factor SOX2 in cancer stem cells suppresses growth and metastasis of lung cancer. *British Journal of Cancer*.

[8] Yoon C-H, Kim M-J, Kim R-K (2012). c-Jun N-terminal kinase has a pivotal role in the maintenance of self-renewal and tumorigenicity in glioma stem-like cells. *Oncogene*.

[9] Ibrahim EE, Babaei-Jadidi R, Saadeddin A (2012). Embryonic NANOG activity defines colorectal cancer stem cells and modulates through AP1- and TCF-dependent mechanisms. *Stem Cells*.

[10] Okada S, Fukuda T, Inada K, Tokuhisa T (1999). Prolonged expression of c-fos suppresses cell cycle entry of dormant hematopoietic stem cells. *Blood*.

[11] Reynolds A, Leake D, Boese Q, Scaringe S, Marshall WS, Khvorova A (2004). Rational siRNA design for RNA interference. *Nature Biotechnology*.

[12] Toloudi M, Apostolou P, Chatziioannou M, Papasotiriou I (2011). Correlation between cancer stem cells and circulating tumor cells and their value. *Case Reports in Oncology*.

[13] Livak KJ, Schmittgen TD (2001). Analysis of relative gene expression data using real-time quantitative PCR and the 2-ΔΔCT method. *Methods*.

[14] Wu XZ (2008). Origin of cancer stem cells: the role of self-renewal and differentiation. *Annals of Surgical Oncology*.

[15] Pan G, Thomson JA (2007). Nanog and transcriptional networks in embryonic stem cell pluripotency. *Cell Research*.

[16] Hattori N, Imao Y, Nishino K (2007). Epigenetic regulation of Nanog gene in embryonic stem and trophoblast stem cells. *Genes to Cells*.

[17] Boer B, Cox JL, Claassen D, Mallanna SK, Desler M, Rizzino A (2009). Regulation of the Nanog gene by both positive and negative cis-regulatory elements in embryonal carcinoma cells and embryonic stem cells. *Molecular Reproduction and Development*.

[18] Das S, Jena S, Levasseur DN (2011). Alternative splicing produces nanog protein variants with different capacities for self-renewal and pluripotency in embryonic stem cells. *The Journal of Biological Chemistry*.

[19] Rodda DJ, Chew J-L, Lim L-H (2005). Transcriptional regulation of Nanog by OCT4 and SOX2. *The Journal of Biological Chemistry*.

[20] Tani Y, Akiyama Y, Fukamachi H, Yanagihara K, Yuasa Y (2007). Transcription factor SOX2 up-regulates stomach-specific pepsinogen A gene expression. *Journal of Cancer Research and Clinical Oncology*.

[21] Zhu Z, Wu G, Wei H (2012). Investigation of the permeability and optical clearing ability of different analytes in human normal and cancerous breast tissues by spectral domain OCT. *Journal of Biophotonics*.

[22] Mani SA, Guo W, Liao M-J (2008). The epithelial-mesenchymal transition generates cells with properties of stem cells. *Cell*.

[23] Kalluri R, Weinberg RA (2009). The basics of epithelial-mesenchymal transition. *The Journal of Clinical Investigation*.

[24] Glover JNM, Harrison SC (1995). Crystal structure of the heterodimeric bZIP transcription factor c-Fos-c Jun bound to DNA. *Nature*.

[25] Yang S-R, Cho S-D, Ahn N-S (2005). The role of p38 MAP kinase and c-Jun N-terminal protein kinase signaling in the differentiation and apoptosis of immortalized neural stem cells. *Mutation Research*.

[26] Wo Y, Zhu D, Yu Y, Lou Y (2008). Involvement of NF-*κ*B and AP-1 activation in icariin promoted cardiac differentiation of mouse embryonic stem cells. *European Journal of Pharmacology*.

[27] Steidl U, Rosenbauer F, Verhaak RGW (2006). Essential role of Jun family transcription factors in PU.1 knockdown-induced leukemic stem cells. *Nature Genetics*.

[28] Mruthyunjaya S, Rumma M, Ravibhushan G, Anjali S, Padma S (2011). c-Jun/AP-1 transcription factor regulates laminin-1-induced neurite outgrowth in human bone marrow mesenchymal stem cells: role of multiple signaling pathways. *FEBS Letters*.

